# DFT Analysis of Hole Qubits Spin State in Germanium Thin Layer

**DOI:** 10.3390/nano12132244

**Published:** 2022-06-29

**Authors:** Andrey Chibisov, Maxim Aleshin, Mary Chibisova

**Affiliations:** Computing Center, Far Eastern Branch of the Russian Academy of Sciences, 680000 Khabarovsk, Russia; aleshin.m.s@pnu.edu.ru (M.A.); omariya2003@yandex.ru (M.C.)

**Keywords:** hole qubit, 2D germanium, quantum states, density functional theory, atomic structure

## Abstract

Due to the presence of a strong spin–orbit interaction, hole qubits in germanium are increasingly being considered as candidates for quantum computing. These objects make it possible to create electrically controlled logic gates with the basic properties of scalability, a reasonable quantum error correction, and the necessary speed of operation. In this paper, using the methods of quantum-mechanical calculations and considering the non-collinear magnetic interactions, the quantum states of the system 2D structure of Ge in the presence of even and odd numbers of holes were investigated. The spatial localizations of hole states were calculated, favorable quantum states were revealed, and the magnetic structural characteristics of the system were analyzed.

## 1. Introduction

Currently, there is active study into solid-state semiconductor materials that can be used to develop quantum computers. Loss and DiVincenzo [1] proposed a quantum computation model based on the spins of electrons enclosed in quantum dots. Later, the so-called DiVincenzo criteria were formulated [2]; it imposes certain requirements on the qubit system. According to these criteria, the system must be scalable and well characterized. Before starting any type of computation, the qubit states must be initialized with a reasonable speed, which is essential for quantum error correction. The system must have a sufficiently long coherence time for the qubit states. Reading computational results should be carried out without affecting neighboring qubits and, as a result, the entire quantum computing system. The first successful application of spin qubits in semiconductors was realized in gallium arsenide (GaAs) [3]. However, one of the main drawbacks of Groups III to V elements is the spin decoherence caused by surrounding nuclear spins. At the same time, silicon is characterized by much less hyperfine interactions, since it consists mainly of ^28^Si atoms with zero nuclear spin that can be isotopically purified. The authors of [4] achieved coherence times on the order of one second for isotopically purified silicon. At the same time, for the gate operation to be fast and fully electrically controllable, a spin–orbit interaction is required, which is absent in the electrons of silicon. Maurand R. et al. [5] showed that holes have the necessary spin–orbit interaction. In theoretical studies [6] it has also been shown that not only filled states of the conduction band but also vacant states of the valence band are promising for the realization of spin qubits. For these reasons, it makes more sense to use structures with holes to produce spin qubit systems. Compared with other semiconductors, germanium has a stronger and more controlled spin–orbit coupling. [7,8,9,10]. Theoretical studies show that, near the Gamma point, the states in the ceiling of the valence band of Ge are well-described by the Luttinger–Kohn Hamiltonian [11,12], whose eigenvalues can be grouped into states with heavy-hole (HH) and light-hole (LH), with values of spin projections on the direction of motion equal to ±3ℏ/2 and ±ℏ/2, respectively. There are several candidate materials for the design of quantum calculators. Such materials can be planar Ge/SiGe heterostructures [13,14], germanium hut wires (HW) [15,16], and germanium core–shell nanowires (NW) [17,18]. HW germanium nanowires grown on silicon surfaces are of the greatest interest. It has been shown that, during the anisotropic growth of Ge on a Si(001) substrate, the quantum dot clusters are pulled along the [001] or [010] Si directions [19]. For the HW structure, the spin relaxation and phase mismatch times were measured, and a single qubit spin control operation was performed. It was also shown that Ge {105} facet formation plays a key role in determining the stability and homogeneity of nanowires [10,16,20,21].

However, all studies carried out so far are purely experimental in nature; there are no works that theoretically describe the behavior of qubits in the proposed germanium-based quantum systems. Thus, the literature data on germanium nanowires are mainly devoted to the description of their production technologies and experimental studies of the behavior of quantum dots in them. Our work is devoted to a detailed analysis of quantum states of the two-dimensional structure system of germanium in the presence of even and odd numbers of holes. The spatial localizations of hole states are investigated in detail, advantageous quantum states are identified, and the magnetic structural characteristics of the system are analyzed.

## 2. Computational Details

All calculations of the atomic structures, their total energy, and charge distribution were performed using the Quantum ESPRESSO software package [22]. The ultrasoft, fully relativistic form of the pseudopotential for germanium within the generalized gradient Perdew–Burke–Ernzerhof approximation in the spin–orbit interaction approximation was used. During the relaxation of the atomic structures of the unit cell and the germanium slab, all atoms were given complete freedom. For structural relaxation, we used the BFGS quasi-newton algorithm [22]. Special sets of k-points were used to sample the Brillouin zone. A 6 × 6 × 6 set was used for the Ge unit cell, and a 2 × 6 × 1 set was used for the ultrathin germanium layer. The cut-off energy of the plane waves was 680.28 eV. The values of the interatomic forces, after structural relaxation, did not exceed the value of 0.026 eV/Å. The atomic geometry and distribution of the charge and magnetic characteristics of the structures were analyzed using the Vesta software package [23].

## 3. Results

**Figure 1:**
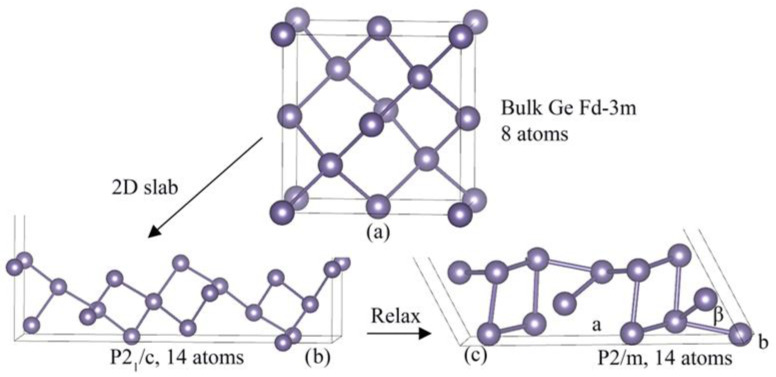
Structure of bulk germanium Fd-3m (**a**), original, unrelaxed structure of the 2D P2_1_/c layer with direction (105) cut from the bulk structure (**b**), relaxed structure of the 2D P2/m layer (**c**).

After the full atomic relaxation of the unit cell of bulk germanium with Fd-3m symmetry consisting of eight Ge atoms [24], we obtained the following cell parameters: *a* = *b* = *c* = 5.616191 Å (Figure 1a). The experimental data for this structure are *a* = *b* = *c* = 5.657820(5) Å [25]. The quasi-two-dimensional structure of germanium with direction (105) was constructed from a bulk cubic cell with the number of atomic layers equal to three and with 14 Ge atoms. The symmetry of the non-relaxed slab structure corresponded to the monoclinic P2_1_/c group with a unit cell basis equal to *a* = 14.31850 Å, *b* = 5.61620 Å and the angle *β* equal to 128.89° (Figure 1b). After full relaxation, the 2D atomic layer remains in the monoclinic structure, but is transformed into the P2/m symmetry with cell parameters equal to *a* = 11.4742 Å, *b* = 4.3403 Å, *β* = 81.53° (Figure 1c). Thus, we see that, during the relaxation of the atomic structure, the cell parameters shrink, with the parameter *a* decreasing by almost 20% and the parameter *b* decreasing by almost 23%. However, since there is a transformation of the angle *β*, there is a corresponding increase in the interatomic distance from 2.463 Å to 2.660–2.730 Å. A similar atomic structure, in the so-called J-germanium phase, was recently theoretically predicted in another paper [26].

**Figure 2:**
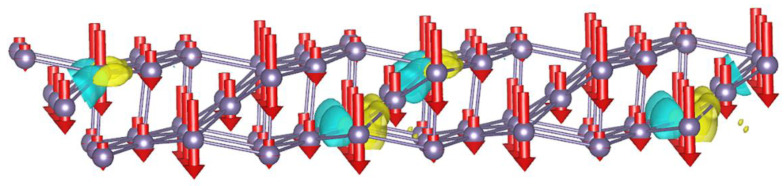
The localization of hole states (one hole) in the germanium with quantum state |1˃, with a spin down direction *s* = −1, is the most energetically advantageous state by 0.72 μeV compared with the state |0˃, with a spin up *s* = +1. The directions of the downward spins *s* = −1 are indicated by red arrows.

**Figure 3:**
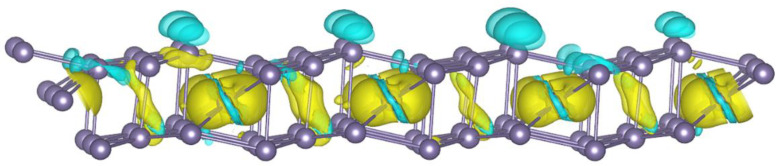
Distribution of the difference in magnetization in the 2D layer of germanium in the presence of a single hole. Yellow indicates states corresponding to |0˃ and blue indicates states corresponding to |1˃.

Then, as a model of a hole qubit in a 2D layer of germanium, a hole was created (a lack of one electron). For this purpose, one electron was removed from the structure, resulting in the formation of a hole in its place. Figure 2 shows the hole state distribution for one hole in the 2D germanium structure with an isosurface level equal to 0.004 (marked in light green). These states were determined by the difference in the bulk charge densities. The neutral system charge density was subtracted from the charge density of the charged system (with a hole present). The hole localization states for systems with two and three holes were determined in the same way. The yellow color in the figure represents the states with increasing charge density; these localizations occur as a result of atomic shifts during the hole formation in the structure. This distribution, which characterizes the hole state, is located in the center of the structure. We see from the figure that similar mirror hole states are observed closer to the edge of the structure. These mirror states are located 11.4742 Å away from each other. Thus, such states arise due to the presence of the magnetic space group P2b2/m in the 2D germanium structure under consideration, which imposes conditions for the translation of magnetic states through the two cell parameters 2*b* along the *Y* axis {2′010 |0 1 0} [27,28]. Indeed, our calculated structure of germanium with P2/m symmetry and cell parameters equal to *a* = 11.4742 Å, *b* = 4.3403 Å, *β* = 81.53° and shown on Figure 1c can be transformed into a triclinic structure with an elementary basis of seven atoms and parameters equal to *a* = 4.3403 Å, *b* = 5.7371 Å, *α* = 81.53°. Then, the distance of 11.4742 Å to which the magnetic state is translated corresponds exactly to twice the value of the parameter b. The difference between the complete magnetic states for the ±1 spin directions is 0.72 μeV. Thus, we see that, for a single-hole qubit in germanium, the quantum state |1˃ with a spin down direction *s* = −1 is the most energetically advantageous state compared to the |0˃ state (i.e., with a spin up direction *s* = +1). The magnetization corresponding to the |1˃ states is more localized on the surface atoms of the 2D germanium layer and the |0˃ states in the center of the structure (Figure 3).

**Figure 4:**
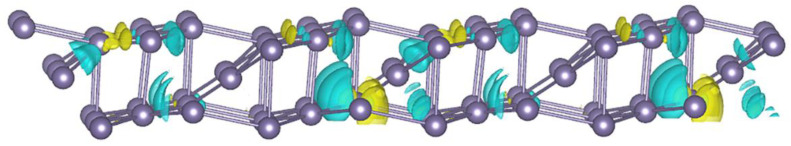
Localization of hole states for two holes in the 2D germanium.

The formation of two holes in the germanium structure leads to the mutual destruction of their magnetic components such that the total magnetization will be zero. Figure 4 shows the localization of hole states for two holes in the 2D germanium structure with an isosurface level equal to 0.004. Thus, an even number of holes does not lead to total magnetization; this result is consistent with the experimental results [20]. With an even number of holes, the most favorable state is the ground singlet state, i.e., with different spin directions.

**Figure 5:**
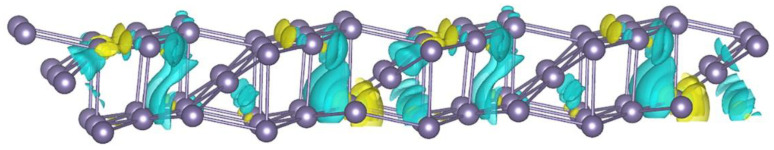
Localization of hole states for three holes in the 2D germanium.

The formation of three holes in the germanium structure, i.e., an odd number of them, results in the quantum state |0˃, with an upward spin direction *s* = +1, becoming the most advantageous state at 0.14 μeV compared to the |1˃ state. This is because, with three holes, the first two occupy a favorable ground singlet state in one orbital, and the third must occupy another higher orbital [20]. As a result, there is Coulomb repulsion between the two holes occupying the singlet state and the third [20,29,30], so a system with three holes is easier to transfer between the |0˃ and |1˃ states compared to a system with a single hole. This would require only 0.14 μeV. Figure 5 shows the localization of hole states for three holes in the germanium structure.

## 4. Conclusions

The atomic and electronic structure of a 2D germanium layer with a crystallographic direction (105) was studied in detail. Spatial localizations of the hole qubit states were investigated, advantageous quantum states were identified, and an analysis of the magnetic structural characteristics of the system was given. It is shown that, for a nanoscale germanium layer with a thickness of 0.27 nm, its atomic structure is transformed into a structure with a monoclinic spatial group with P2/m symmetry. This transformation is accompanied by an increase in the interatomic distance. We analyzed the quantum states of the hole qubits in the system in the presence of an even and odd number of holes. The results show that, for a single hole, the advantageous quantum state at 0.72 μeV is the |1˃ state, with a spin down direction *s* = −1, compared to the |0˃ state, with a spin up direction *s* = +1. An even number of holes in the system does not result in full magnetization. The formation of three holes causes the quantum state |0˃, with a spin up direction *s* = +1, to become the most advantageous state at 0.14 μeV compared to the state |1˃, so a system with three holes is easier to transfer between the quantum states |0˃ and |1˃ compared to a system with one hole. The paper shows that hole qubits are characterized by the condition of the translation of their magnetic states through two cell parameters 2*b* along the *Y* axis. We are confident that our theoretical results will be relevant and promising for use by technologists and experimentalists in the design and study of quantum computing systems based on hole qubits.

## Data Availability

The data presented in this study are available on request from the corresponding author.
